# Antiviral and immunomodulatory effects of Siji antiviral mixture against H9N2 avian influenza virus infection in chickens

**DOI:** 10.1016/j.psj.2026.107336

**Published:** 2026-06-24

**Authors:** Yanjiao Liang, Jingting Yang, Changmao Jian, Miaoxiang Zhang, Kewei Chen, Junwei Yang, Ping Luo, Meilan Mo, Tianchao Wei, Teng Huang, Jianni Huang

**Affiliations:** a College of Animal Science and Technology, Guangxi University, Nanning, 530004, China; b Guangxi Key Laboratory of Animal Breeding, Disease Control and Prevention, Nanning, 530004, China; c Guangxi Zhuang Autonomous Region Engineering Research Center of Veterinary Biologics, Nanning, 530004, China

**Keywords:** H9N2 avian influenza virus, Siji antiviral mixture, Antiviral, Replication, Inflammation

## Abstract

The H9N2 subtype avian influenza virus (AIV) is common in poultry and poses significant risks to both the poultry industry and public health. Current control strategies for H9N2 AIV predominantly rely on vaccination; however, these approaches are often undermined by the continuous antigenic drift of hemagglutinin under the pressure of antibodies. This study evaluated the antiviral efficacy of Siji Antiviral Mixture (SAM) against H9N2 infection in specific pathogen-free chickens. Compared with the infected control group, SAM significantly reduced viral loads in multiple tissues, including liver, spleen, lung, kidney, brain, and trachea, demonstrating its direct antiviral activity. Further analysis revealed that SAM modulated H9N2-induced inflammation by downregulating excessive innate immune responses. Specifically, SAM significantly enhanced the expression of antiviral effectors (OASL and MX-1) in the lungs during the early stages of viral infection, while attenuating the production of inflammatory cytokines (IL-6, IL-10) in the later stages. Additionally, SAM regulated the expression levels of pattern recognition receptors (MDA5, TLR3, and TLR7), thereby preventing excessive immune responses. These findings indicate that SAM possesses both antiviral and immunoregulatory effects, highlighting its potential as an effective anti-AIV agent for the management of H9N2 AIV infection.

## Introduction

Avian influenza viruses (AIVs), members of the genus influenza A virus within the family *Orthomyxoviridae*, are classified into highly pathogenic avian influenza viruses (HPAIVs) and low pathogenic avian influenza viruses (LPAIVs) based on their pathogenicity in chickens (Webster et al., 1992; Cui et al., 2020; Tian et al., 2021). The H9N2 subtype AIV is an LPAIV that is widespread among domestic poultry and wild birds (Peacock et al., 2019; Carnaccini and Perez, 2020; Guo et al., 2021). Moreover, H9N2 AIV acts as a genetic donor, contributing its internal genes to other AIV subtypes, such as H3N8, H7N9, H10N8, and H5N6 (Gao et al., 2013; Chen et al., 2014; Bhat et al., 2022; Peng et al., 2022; Yang et al., 2022). This genetic exchange enables these viruses to acquire the capability to bind to mammalian receptors, facilitating cross-species transmission and the infection of humans and other mammals. Consequently, H9N2 AIV poses a significant threat to both the poultry industry and public health.

H9N2 AIV can replicate in the digestive or respiratory tracts of poultry, typically causing mild respiratory symptoms (Peacock et al., 2019; Guo et al., 2021). Infection with H9N2 AIV induces inflammatory responses in birds, and the virus can be isolated from various organs, including the pancreas, lungs, brain, spleen, cloaca, and intestines (Nang et al., 2011). However, H9N2 AIV infection is often associated with co-infection or secondary infection involving other viruses or bacteria, which exacerbates clinical symptoms and increases mortality rates in poultry flocks (Erfan et al., 2019; Kong et al., 2022).

Upon viral infection, host cells initiate innate immune responses through recognizing pathogen-associated molecular patterns (PAMPs) by pattern recognition receptors (PRRs) (Chen et al., 2013; Iwasaki and Pillai, 2014). In chickens, the primary PRRs responsible for sensing AIV RNA are melanoma differentiation-associated protein 5 (MDA5), Toll-like receptor 3 (TLR3), and TLR7 (Iqbal et al., 2005; Philbin et al., 2005; Karpala et al., 2008; Karpala et al., 2011; Hayashi et al., 2014). This recognition triggers the production of type I interferons (IFNs) and pro-inflammatory cytokines. Secreted type I IFNs subsequently induce the expression of interferon-stimulated genes (ISGs), such as oligoadenylate synthase-like (OASL) and myxovirus resistance (MX-1), which play critical roles in establishing an antiviral state (Haller et al., 2015; He et al., 2024; Schneider et al., 2014; Tag-El-Din-Hassan et al., 2017). However, AIV infection can also induce excessive cytokine production, leading to a “cytokine storm” in infected tissues (Liu et al., 2021). Cytokines are broadly classified as pro-inflammatory or anti-inflammatory based on their immunomodulatory functions. The magnitude and kinetics of pro-inflammatory cytokine (e.g., IL-6, IL-8, IL-12, and TNF-α) and anti-inflammatory cytokine (e.g., IL-4, IL-10, IL-11, IL-13, and TGF-β) responses are closely associated with viral pathogenicity and the extent of tissue damage (Monastero and Pentyala, 2017; Liu et al., 2021). Previous studies have identified that various cytokines, including IFN-α, IL-6, IL-8, and IL-10, are detected in the lungs, spleen, and cecal tonsils of chickens infected with H9N2 AIV (Nang et al., 2011). Notably, the levels of cytokine induction are directly proportional to the virulence of AIV strain (Xing et al., 2008; Rebel et al., 2011; Vervelde et al., 2013; Kalaiyarasu et al., 2016). Given that the immune regulatory mechanisms are critical in determining the outcome of H9N2 AIV infection, evaluating the impact of antiviral factors on these pathways is necessary.

In China, the primary strategy for preventing and controlling H9N2 AIV involves the use of inactivated vaccines (Wei et al., 2016; Hu et al., 2025). The initial implementation of these vaccines has demonstrated considerable efficacy in curtailing the spread of H9N2 AIV. However, their extended use has facilitated antigenic drift in the HA gene of H9N2 AIV due to immunological pressure, leading to a reduction in the efficacy of traditional inactivated vaccines (Li et al., 2017; Sha et al., 2020). Although novel vaccines, such as oral vaccines, multi-epitope vaccines, and recombinant viral vector vaccines, have exhibited immunogenicity, their long-term clinical safety and efficacy require further validation (Sha et al., 2020). Antiviral drugs represent a potential intervention, but the emergence of viral drug resistance presents a major challenge for current antiviral development (Bonomini et al., 2025). Furthermore, H9N2 AIV infection frequently predisposes hosts to secondary bacterial infections, particularly *Escherichia coli* (*E. coli*), so antibiotics are frequently utilized to manage inflammation associated with H9N2 AIV (Zhang et al., 2020; Liang et al., 2023). However, the widespread use of antibiotics often leads to the emergence of resistant bacterial strains and the presence of drug residues in animal products, posing risks of environmental contamination and threatening human health (Zhang et al., 2020; Liang et al., 2023). It has been proposed that novel antiviral agents are capable of controlling AIV transmission by inhibiting excessive pro-inflammatory responses and reducing tissue damage (Cheng et al., 2018; Chen and Huang, 2018; Kallon et al., 2013; Song et al., 2015).

Traditional Chinese medicine (TCM) offers advantages due to its potential to target both virus and host, thereby effectively suppressing the host’s inflammatory immune response induced by IAV (Ramos and Fernandez-Sesma, 2015; Ma et al., 2023; He et al., 2026). Recently, natural products with inhibitory activity against AIV have been tested and reported in the literature (Chen and Huang, 2018; Zhou et al., 2021; Zhi et al., 2024; Elsherbini et al., 2025). For instance, astragalus polysaccharides have been shown to protect against influenza virus-induced lung injury through modulation of the TLR4/7-MyD88-NF-κB signaling pathway (Chen and Huang, 2018). Baicalin has been found to regulate *Lactobacillus* populations to suppress secondary *E. coli* infection caused by H9N2 AIV and effectively inhibit virus-induced inflammatory responses (Zhang et al., 2020). Song *et al.* showed that sulfated polysaccharides extracted from three species of algae exhibited various diverse anti-AIV activities both *in vitro* and *in vivo* (Song et al., 2015). Cheng *et al.* found that a combination of algal sulfated polysaccharides and a compound extract from traditional Chinese medicine significantly inhibited the proliferation of H9N2 AIV in specific pathogen-free (SPF) chicken embryos (Cheng et al., 2018). Therefore, the development of novel therapeutics from TCM represents a viable alternative strategy against H9N2 AIV infection.

Siji Antiviral Mixture (SAM), a compound-based Chinese medicine, has obtained regulatory approval from the China State Food and Drug Administration and is clinically employed in the treatment of viral conditions, including the common cold, influenza, mumps, and other infectious diseases (Zhang and Qi, 2009; Yao et al., 2019; Yang et al., 2021). The active constituents of SAM are a variety of botanical sources, including *Houttuynia cordata, Schizonepeta tenuifolia, Mentha haplocalyx, Platycodon grandiflorus, Mori folium, Forsythia suspensa, Perilla frutescens leaves, Armeniaca sibirica kernels, Phragmites communis rhizome, Chrysanthemum morifolium*, and *Glycyrrhiza uralensis.* The principal active compounds identified within this formulation are chlorogenic acid, liquiritin, rutin, forsythoside A, isochlorogenic acid A, forsythin, and glycyrrhizic acid (Xiao et al., 2018; Yao et al., 2019; Wang et al., 2025). Recently, its therapeutic scope has been extended to pediatric respiratory infection and hand, foot, and mouth disease (HFMD) (Cao et al., 2012; Chen et al., 2013). However, the therapeutic potential of SAM against H9N2 AIVs in birds remains unknown. In this study, the efficacy of SAM against H9N2 AIV infection was evaluated in SPF chickens by quantifying virus titers and analyzing immune-associated efforts in tissues.

## Materials and Methods

### Ethics statement and facility

All procedures conducted in this study were approved by the biosafety committee of Guangxi University (GXUKE2021-02; 16 March 2021). In addition, all animal-related experiments and experimental protocols were certified by the Animal Ethics Committee of Guangxi University (Guangxi, China), under the Animal Experimental Ethics Review approval number “GXU-2025-199”.

### Virus

The strain A/Duck/Dongguan/0601Y/2021 (H9N2), isolated from a live poultry market, was stored at the Avian Disease Laboratory, College of Animal Science and Technology, Guangxi University.

### Preparation of Chinese medicine

First, steam distillation was performed on *Houttuynia cordata, Schizonepeta tenuifolia*, and *Mentha haplocalyx*. A total of 700 mL of the distillate was collected and set aside for later use. The herbal residues from the distillation were mixed with the remaining eight herbs (*Platycodon grandiflorus, Mori folium, Forsythia suspensa, Perilla frutescens leaves, Armeniaca sibirica kernels, Phragmites communis rhizome, Chrysanthemum morifolium*, and *Glycyrrhiza uralensis*) until evenly blended. Water was added, and the mixture was decocted twice, for 2 hours each time. The decoctions were combined and filtered, and then the filtrate was combined with the liquid remaining after the aforementioned volatile oil extraction.

The combined liquid was concentrated to a clear paste with a relative density of 1.10 (at 60°C). Ethanol was added to the clear paste until the alcohol content reached 60%. The mixture was allowed to stand for 24 hours and then filtered. The ethanol was recovered from the filtrate, and the liquid was concentrated until no alcohol odor remained. To this concentrate, 100 g of sucrose and 3 g of sodium benzoate were added. The mixture was brought to a boil and then allowed to stand and filter.

The resulting liquid was mixed with the 700 mL of distillate collected earlier and stirred until homogeneous. The total volume was adjusted to 1000 mL. The final mixture was filtered and packaged to obtain the finished product. The drug dose conversion coefficients between chickens and other experimental animals were calculated based on the body weight and body surface area of the chickens. The K value calculated from the chicken Meeh-Rubner formula was 9.24 ± 1.03. The mg/kg–mg/m² transfer factor was 10.00 ± 1.36, and the relative ratio of the unit of surface area and body weight was 0.30 ± 0.044 (Li et al., 2018).

### SPF chicken experiments

Five-week-old SPF chickens and 9–11-day-old SPF chicken embryos were purchased from Xinxing Dahuanong Co., Ltd. The animal experiments were performed according to previous studies (Kallon et al., 2013; Shang et al., 2020; Zhi et al., 2024). A total of 90 SPF chickens were randomly divided into six groups (n = 15 per group): pre-addition of SAM low-dose group(G1), pre-addition of SAM middle-dose group (G2), post-addition of SAM middle-dose group (G3), post-addition of SAM high-dose group (G4), infected group (G5), and control group (G6) (Table 1). The treatments for each group were as follows:

**Table 1:** The treatment for the experimental groups in this study.

Group No.	Group Designation	Administration Route	SAM Dosage	Administration Period
G1	Pre-addition of SAM low-dose group	Oral gavage	1.53 g/kg/day	1 to 7 days pre-infection
G2	Pre-addition of SAM middle-dose group	Oral gavage	3.06 g/kg/day	1 to 7 days pre-infection
G3	Post-addition of SAM middle-dose group	Oral gavage	3.06 g/kg/day	2 to 8 days post-infection
G4	Post-addition of SAM high-dose group	Oral gavage	6.12 g/kg/day	2 to 8 days post-infection
G5	Infected group	Oral gavage	1 mL PBS/day	2 to 8 days post-infection
G6	Control group	Oral gavage	1 mL PBS/day	7 days pre-infection to 8 days post-infection

**Fig. 1:**
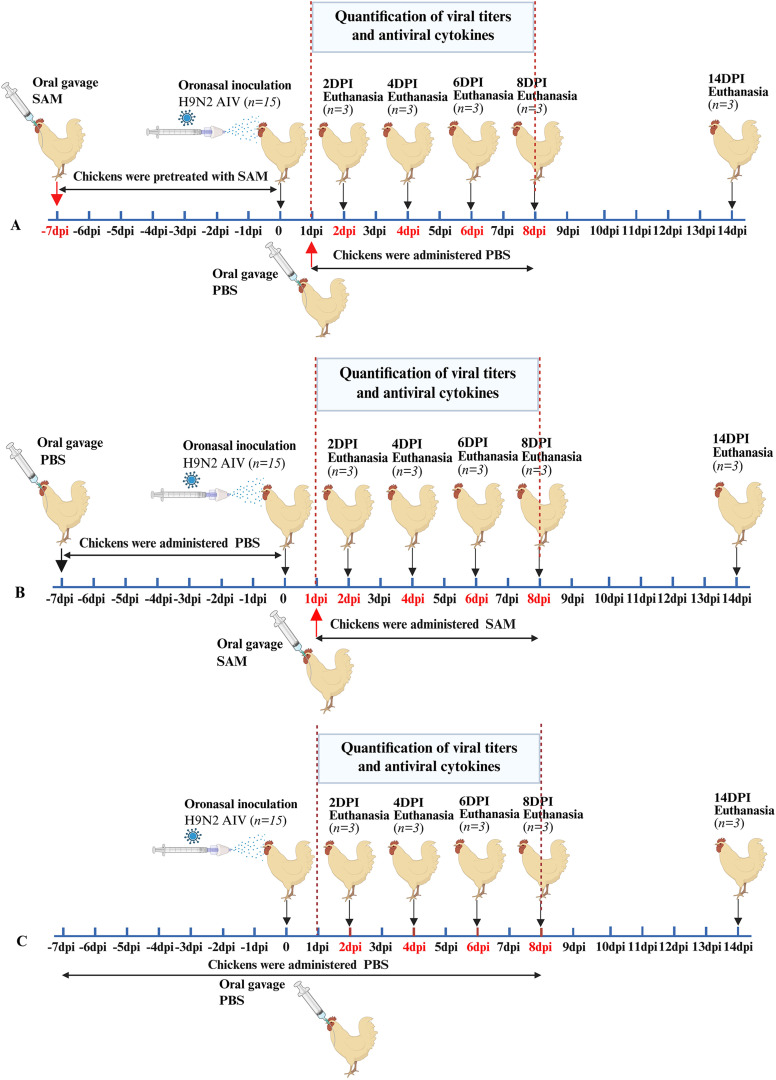
The experimental design for evaluating the therapeutic and preventive efficacy of SAM against H9N2 AIV infection. (A) Preventive groups. (B) Therapeutic groups. (C) Infected group.

The experimental design included six groups to assess the preventive and therapeutic efficacy of SAM. The preventive groups were the pre-addition of SAM low-dose group (G1) and pre-addition of SAM middle-dose group (G2), while the therapeutic groups included the post-addition of SAM middle-dose group (G3) and post-addition of SAM high-dose group (G4). Each chicken in the preventive, therapeutic, and infected groups was inoculated intranasally with 10^8^ EID_50_/0.2 mL of H9N2 AIV at 0 day post-infection (DPI) (Fig. 1). Concurrently, each chicken in the control group was challenged with 0.2 mL PBS via the same route. After 1 day post-infection, the chickens in the therapeutic groups were administered SAM via oral gavage at dosages of 3.06 g/kg (G3 group) or 6.12 g/kg (G4 group) once daily until 8 DPI. The chickens in the preventive groups were pretreated with SAM at dosages of 1.53 g/kg (G1 group) or 3.06 g/kg (G2 group) for 7 consecutive days. While the chickens in the infected (G5) and control groups (G6) received an oral gavage of 1 mL PBS (Fig. 1).

Subsequently, chickens in all groups were observed daily for clinical signs, including mortality, appetite, and mental status. At 2, 4, 6, and 8 DPI, three chickens from each group were euthanized for necropsy, and tissue samples, including liver, spleen, lung, kidney, brain, and trachea, were collected (Fig. 1). One gram of each tissue was homogenized in 1 mL PBS using a tissue homogenizer. After centrifugation, the supernatant was serially diluted 10-fold. Each dilution was inoculated into three 9–11-day-old SPF chicken embryos (0.1 mL/embryo), which were incubated at 37°C for 48 hours. Virus infection was determined by hemagglutination (HA) assay, and the viral titer was calculated using the Reed-Muench method (Brown, 1964), with results expressed as log₁₀ EID₅₀/g.

### The mRNA expression of immune-related genes in lungs and spleen of SPF chickens

At 2, 4, 6, and 8 DPI, lung and spleen tissue were collected from three chickens per group. A total of 20 mg of each tissue sample was homogenized and centrifuged at low temperature. Total RNA was extracted using an RNA extraction kit (Feijie, China), and 1 μg of total RNA from each sample was reverse-transcribed into cDNA. Real-time quantitative PCR (RT-qPCR) was performed using 2×SYBR Green qPCR Master Mix (Bimake, Shanghai, China) to quantify the mRNA expression levels of immune-related genes. The primers used in RT-qPCR are provided in Table 2. The amplification protocol consisted of an initial denaturation at 95°C for 2 min, followed by 40 cycles of 95°C for 5 s and 60°C for 40 s. The Ct values were recorded for subsequent analysis.

**Table 2:** The primers used for RT-qPCR in this study.

Name	Sequence of oligonucleotide (5’-3’)	Target gene
TLR3-F	CTCTATTCCTTGCTGGAA	Chicken TLR3
TLR3-R	CTCGGTCAGATTTTCAGGAT	
TLR7-F	AGAAGGTGTTAGCCACGTG	Chicken TLR7
TLR7-R	TCAGATTCTTGAAGAACGA	
MDA5-F	ATGTCGGAGGAGTGCCGAGAC	Chicken MDA5
MDA5-R	TTAATCTTCATCACTTGAAGGA	
IFN-β-F	CCAGCTCCTTCAGAATACG	Chicken IFN-β
IFN-β-R	GAGGCTGTGGCGTGTGCGGTC	
MX-1-F	CATGGTCCAACTTCAGCTC	Chicken MX-1
MX-1-R	ATCCTTGTCCTCTTCTCTGTC	
OASL-F	GACAGCCGGGACAGACCGC	Chicken OASL
OASL-R	CACTTCACAAAGTTCTTCTGC	
IL-6-F	GCTGGCTTCGACGAGGAGA	Chicken IL-6
IL-6-R	ATGACCACTTCATCAGGATT	
IL-10-F	TGGCTGCATGCACCCTGCC	Chicken IL-10
IL-10-R	AGCGCAGCATCTCTGACACAG	
TNF-α-F	CCGCCCAGTTCAGATGAG	Chicken TNF-α
TNF-α-R	GGCATTGCAATTTGGACA	
β-actin-F	CCCAGACATCAGGGTGTGAT	β-actin
β-actin-R	GGCTTTGGGGTTCAGGGGAG	

### Statistical analysis

The viral titers and the mRNA expression of immune-related factors are shown as mean ± SD (n = 3). Statistical analysis was performed using a one-way ANOVA in GraphPad Prism software (version 10.0; GraphPad Software, San Diego, CA, USA). Significance levels were indicated as follows: #*p* < 0.05, *P < 0.05; ##*P* < 0.01, **P < 0.01; ###*P* < 0.001, ***P < 0.001; ####*P* < 0.0001, ****P < 0.0001. The notation “ns” denotes no significant difference.

## Results

### Effect of SAM on H9N2 AIV replication in SPF chicken tissue

**Fig. 2:**
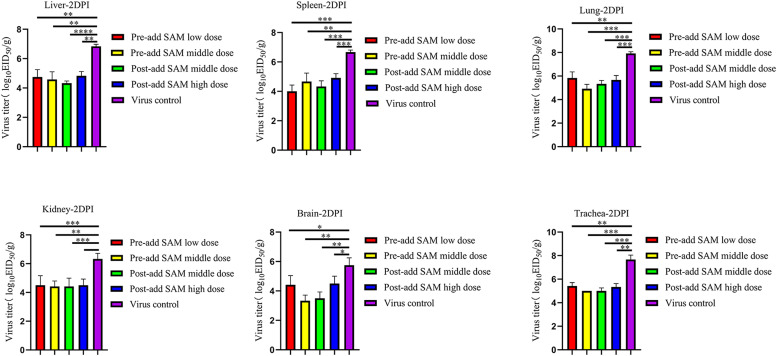

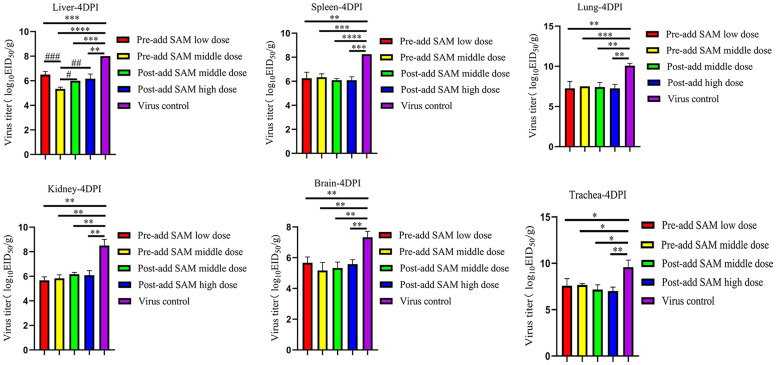

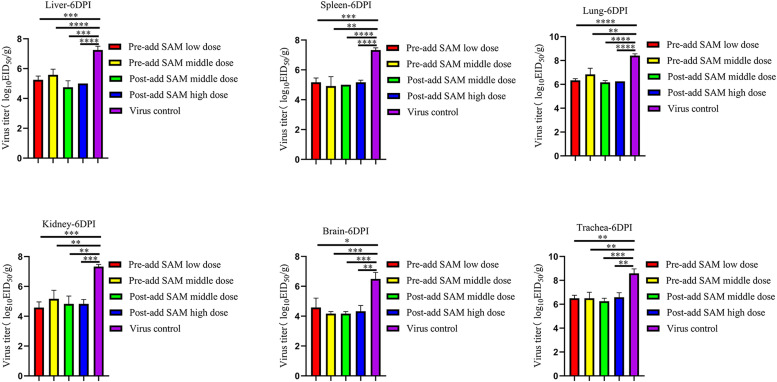

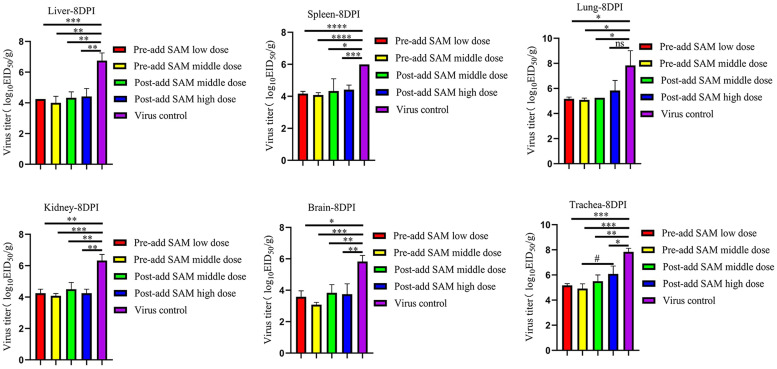
SAM inhibits H9N2 AIV replication in various tissues of SPF chickens at 2 DPI (A), 4 DPI (B), 6 DPI (C), and 8 DPI (D). Viral titers were determined by HA assay and calculated using the Reed-Muench method, expressed as log₁₀EID₅₀/g. Data are presented as mean ± SD (n = 3). Statistical significance was analyzed using one-way ANOVA followed by Tukey’s post-hoc test. Significant differences between the SAM-treated groups and infected group are indicated as *P < 0.05, **P < 0.01, ***P < 0.001, and ****P < 0.0001. Significant differences between the G1, G2, G3, and G4 groups are denoted as #*P* < 0.05, ##*P* < 0.01, ###*P* < 0.001, and ####*P* < 0.0001.

To investigate the antiviral efficacy of SAM against H9N2 AIV infection, viral titers were assessed in various tissues, including the brain, spleen, kidney, lung, liver, and trachea, across the G1–G5 groups. The results showed that infected chickens exhibited mild respiratory symptoms and ruffled feathers, but no mortality was observed. H9N2 AIV demonstrated effective replication across multiple tissues in infected chickens, with peak viral titers occurring at 6 DPI. The viral titers in tissues from SAM-treated groups were significantly lower than those in the group only infected with H9N2 AIV at 2 DPI (Fig. 2A), 4 DPI (Fig. 2B), 6 DPI (Fig. 2C), and 8 DPI (Fig. 2D).

Interestingly, in the SAM-treated groups, viral titers in livers from the G2 group were significantly lower than those in the G1, G3, and G4 groups at 4 DPI (Fig. 2B). Similarly, viral titers in the trachea were lower in the G2 group at 8 DPI compared with the other groups (Fig. 2D). These results indicate that SAM significantly inhibited H9N2 AIV replication in the tissues of SPF chickens, with the middle dose providing the optimal protective and therapeutic effect.

### Effect of SAM on immune-related factors in tissues of H9N2 AIV-infected SPF chickens

#### Effect of SAM on expression of PRRs induced by H9N2 AIV

**Fig. 3:**
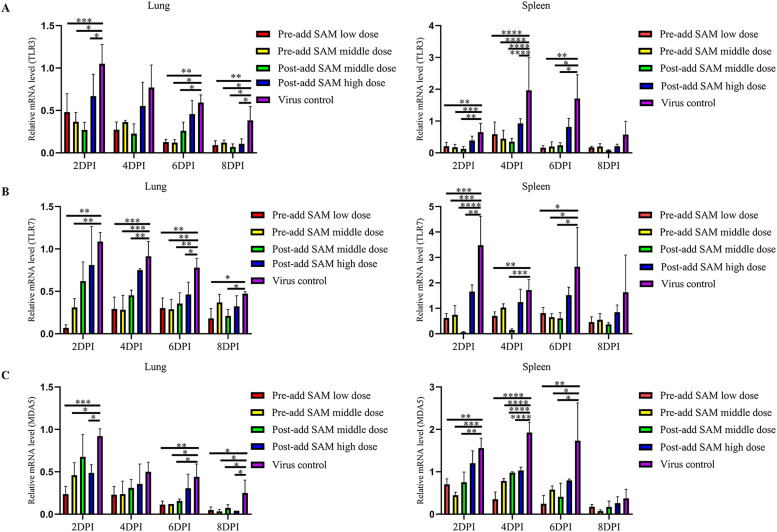
(A–C) SAM maintains moderate expression of PRRs in the lung and spleen tissue of SPF chickens. The relative mRNA expression levels of TLR3 (A), TLR7 (B), and MDA5 (C) in the lung and spleen were quantified using RT-qPCR. Tissues were collected from three chickens per group at 2, 4, 6, and 8 DPI. Total RNA (1 μg per tissue) was extracted and reverse-transcribed into cDNA. Each individual cDNA sample was assayed in triplicate. The mRNA expression of each tissue was normalized to β-actin using the 2^−ΔΔCt^ method and was expressed relative to the corresponding tissue in the control group (G6). Data are shown as mean ± SD (n=3). Statistically significant differences between the SAM-treated and infected groups were determined by one-way ANOVA followed by Tukey’s post-hoc test. Significance levels are represented by *P < 0.05, **P < 0 .01, ***P < 0.001, and ****P < 0.0001. The notation “ns” denotes no significance.

In the infected group, H9N2 infection induced elevated mRNA expression of pattern recognition receptors MDA5, TLR3, and TLR7 in lung tissue during the early stage of infection. The mean mRNA expression levels of MDA5, TLR3, and TLR7 in lung tissues from all SAM treatment groups (both preventive and therapeutic groups) were lower than those in the infected group (Fig. 3A–C). In particular, the preventive groups (G1 and G2) exhibited significantly reduced mRNA levels of MDA5 and TLR3 compared to the infected group at 2, 6, and 8 DPI. Similarly, the G3 group demonstrated a significant reduction at 6 and 8 DPI (Fig. 3A and C). In comparison to the infected group, TLR7 mRNA levels in the G1 and G2 groups were significantly decreased at 2, 4, and 6 DPI. While in the G3 group, significant reductions of TLR7 mRNA were observed at 4, 6, and 8 DPI (Fig. 3B).

In the spleen tissue, the expression of PRRs mRNA was also elevated during the early stage in the infected group. All SAM treatment groups exhibited lower mRNA levels for MDA5, TLR3, and TLR7 compared to the infected group (Fig. 3A–C). Notably, the G1 and G3 groups showed significantly lower mRNA levels of MDA5 and TLR3 at 2, 4, and 6 DPI, whereas the G4 group demonstrated a significant reduction at 6 DPI (Fig. 3A and 3C). These results suggest that H9N2 AIV induces the early expression of PRRs during the initial stages of infection, while Siji Antiviral Mixture appears to sustain moderate expression levels of PRRs, potentially mitigating an excessive immune response.

#### Effect of SAM on Expression of IFN-β and ISGs Induced by H9N2 AIV

**Fig. 4:**
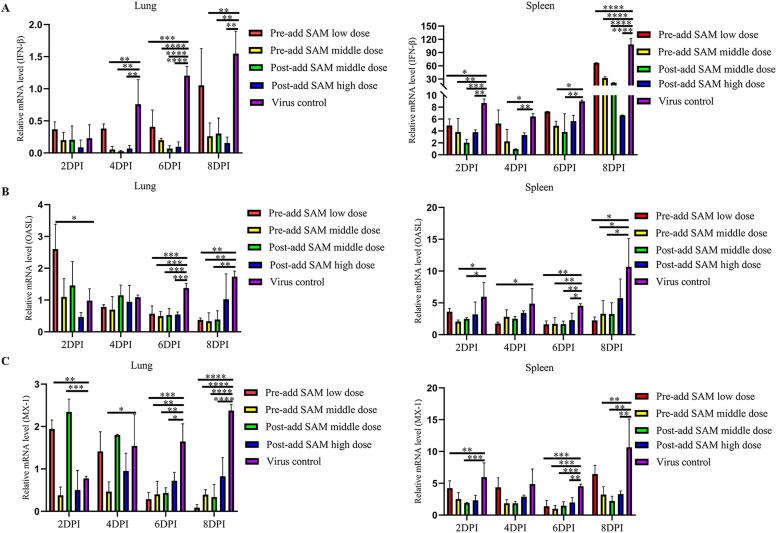
(A–C) SAM modulates the expression of IFN-β and ISGs in the lung and spleen tissue of SPF chickens. The relative mRNA expression of IFN-β (A), OASL (B), and MX-1 (C) in the lung and spleen was quantified using RT-qPCR. Tissues were collected from three chickens per group at 2 DPI, 4 DPI, 6 DPI, and 8 DPI. Each individual cDNA sample was assayed in triplicate. The mRNA expression of each tissue was normalized to β-actin using the 2^−ΔΔCt^ method and was expressed relative to the corresponding tissue in the control group (G6). Data are shown as mean ± SD (n=3). Statistically significant differences between the SAM-treated and infected groups were determined using one-way ANOVA followed by Tukey’s post-hoc test. Significance difference: *P < 0.05, **P < 0.01, ***P < 0.001, and ****P < 0.0001. The notation “ns” denotes not significant.

In the lung tissue, H9N2 infection in the infected group resulted in elevated mRNA expression levels of IFN-β and antiviral factors such as OASL and MX-1 during the later stages of infection. IFN-β mRNA levels in groups treated with SAM were significantly lower than those in the infected group at 4 DPI, 6 DPI, and 8 DPI (Fig. 4A). OASL mRNA levels in the G1 and G3 groups were significantly higher than those in the infected group at 2 DPI, but significantly lower at 6 DPI and 8 DPI. Conversely, OASL mRNA levels in the G2 and G4 groups were significantly reduced compared to the infected group at 8 DPI (Fig. 4B). Similarly, MX-1 mRNA levels in the G1 and G3 groups were significantly higher than those in the infected group at 2 DPI but decreased at 6 DPI and 8 DPI. MX-1 mRNA levels in the G2 and G4 groups exhibited a significant reduction at 6 and 8 DPI (Fig. 4C).

In spleen tissue, the mRNA expression levels of IFN-β, OASL, and MX-1 were markedly increased during the late stages of infection in the infected group. In contrast, all SAM treatment groups exhibited lower mRNA levels for these genes compared to the infected group. IFN-β mRNA levels in G2 and G3 were significantly diminished at 2, 4, 6, and 8 DPI, while the G1 and G4 groups exhibited significant reductions at 2 and 8 DPI (Fig. 4A). OASL mRNA levels in the G2 and G3 groups were significantly lower at 2 and 6 DPI, whereas in the G1 and G4 groups, significant reductions were observed at 6 DPI and 8 DPI (Fig. 4B). MX-1 mRNA levels in the G2, G3, and G4 groups were significantly reduced at 6 and 8 DPI, with G1 showing reductions at 6 DPI and 8 DPI (Fig. 4C). These results indicate that SAM may enhance the expression of OASL and MX-1 during the early stages of H9N2 AIV infection and suppress the overexpression of IFN-β, OASL, and MX-1 in later stages of infection.

#### Effect of SAM on Expression of Inflammatory Cytokines Induced by H9N2 AIV

**Fig. 5:**
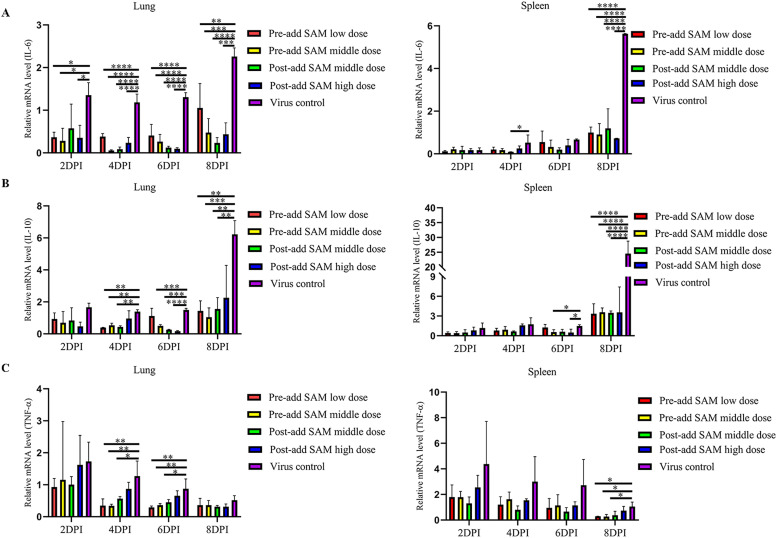
(A–C) SAM inhibits the expression of inflammatory cytokines in the lung and spleen of SPF chickens. The relative mRNA expression of IL-6 (A), IL-10 (B), and TNF-α (C) in the lung and spleen was quantified using RT-qPCR. Tissues were collected from three chickens per group at 2, 4, 6, and 8 DPI. Each individual cDNA sample was assayed in triplicate. The mRNA expression of each tissue was normalized to β-actin using the 2^−ΔΔCt^ method and was expressed relative to the corresponding tissue in the control group (G6). Data are shown as mean ± SD (n=3). Statistically significant differences between the SAM-treated and infected groups for each tissue were determined by one-way ANOVA followed by Tukey’s post-hoc test. Significance difference: *P < 0.05, **P < 0.01, ***P < 0.001, and ****P < 0.0001. The notation “ns” denotes not significant.

The mRNA levels of inflammatory cytokines, including IL-6, IL-10, and TNF-α in lung and spleen tissues, were analyzed using RT-qPCR. In lung tissue, H9N2 AIV infection led to the upregulation of IL-6 and IL-10 during the late stages of infection, while TNF-α expression levels were elevated during the early stages. All SAM treatment groups showed lower mRNA expression levels of IL-6, IL-10, and TNF-α compared to the infected group (Fig. 5A–C). Notably, IL-6 and IL-10 mRNA levels were significantly lower in all SAM treatment groups than in the infected group (Fig. 5A and B). TNF-α mRNA levels in G1, G2, and G3 were significantly reduced at 4 and 6 DPI (Fig. 5C).

In spleen tissue, H9N2 AIV similarly upregulated IL-6 and IL-10 during late infection and increased TNF-α during early infection. IL-6 mRNA levels in all SAM treatment groups were significantly lower than in the infected group at 8 DPI (Fig. 5A). IL-10 mRNA levels in the G1 and G3 groups were significantly reduced at 8 DPI, while those in G2 and G4 were significantly lower at 6 and 8 DPI (Fig. 5B). TNF-α mRNA levels in the G1, G2, and G3 groups were significantly lower than in the infected group at 8 DPI (Fig. 5C).

These findings suggest that H9N2 AIV triggers elevated levels of inflammatory cytokines during the late stages of infection, while Siji Antiviral Mixture could suppress the excessive expression of these cytokines, potentially mitigating tissue damage.

## Discussion

H9N2 AIV is an LPAIV widely circulating in Chinese poultry. It causes significant morbidity, particularly in the presence of co-infection or secondary infection with bacteria or other viruses, resulting in substantial economic losses (Carnaccini and Perez, 2020; Erfan et al., 2019; Kong et al., 2022; Peacock et al., 2019). Currently, there are neither effective preventive measures nor satisfactory antiviral drugs available for the management of H9N2 AIV. Therefore, it’s necessary to develop effective antiviral drugs for both the prevention and treatment of H9N2 AIV infection. In this study, we investigated the antiviral efficacy of SAM on the H9N2 AIV infection. The results demonstrated that SAM significantly inhibited viral replication in multiple tissues at 2, 4, 6, and 8 DPI. Moreover, SAM modulated the expression of PRRs and ISGs while suppressing excessive inflammatory cytokine responses. These findings indicate that SAM possesses both antiviral and immunoregulatory effects, highlighting its potential as an anti-AIV agent against H9N2 AIV infection.

Several natural products have been reported to inhibit H9N2 AIV replication via diverse mechanisms (Elsherbini et al., 2025; Jin et al., 2018; Li et al., 2018; Zhou et al., 2021). For instance, *Zataria multiflora* essential oil reduces clinical signs by inhibiting viral replication in infected birds, whereas Oroxylin A exhibits antiviral effects by inhibition of virus replication and NA activity (Jin et al., 2018; Shayeganmehr et al., 2018). Similarly, Cappariloside A exerts host antiviral effects by modulating interferon signaling pathways and suppressing H9N2-induced inflammation (Li et al., 2018). Additionally, arctiin, a bioactive lignan glycoside, exerts antiviral activity against H9N2 virus through suppressing virus-induced pro-inflammatory cytokines and blocking virus-mediated activation of RIG-I/JNK MAPK signaling (Zhou et al., 2021). SAM is known for its heat-clearing, detoxifying, anti-inflammatory, and antipyretic properties. Previous studies have shown that a combination of SAM and oseltamivir phosphate granules reduces the inflammatory response and promotes the improvement of children’s condition (Li and Zhang, 2009; Liu and Dong, 2021; Tang and Wang, 2021). Consistent with these reports, the present study demonstrated that SAM significantly inhibited viral replication in SPF chickens. However, this study did not include antiviral drugs effective against human influenza, such as oseltamivir and amantadine, which limits the ability to compare the relative efficacy of SAM.

Upon viral infection, chicken MDA5, TLR3, and TLR7 serve as the primary receptors for sensing viral RNA (Karpala et al., 2008; Karpala et al., 2011; Philbin et al., 2005). Chicken MDA5 can potentially compensate for the lack of RIG-I signaling following AIV infection (Karpala et al., 2011; Hayashi et al., 2014). The expression of TLR3 and TLR7 mRNA is rapidly upregulated in the lungs, gut, and bursa of chickens in response to H7N1 LPAIV infection (Cornelissen et al., 2012). Therefore, the activation of the PRRs-mediated signaling pathway exerts a protective effect against tissue injury caused by AIV. Nevertheless, excessive activation of these pathways may lead to harmful inflammation (Arora et al., 2019; Shi et al., 2020; Zhang et al., 2021). In the present study, H9N2 AIV infection was found to significantly upregulate the expression of PRRs during the initial stages of infection. Interestingly, PRRs expression in the SAM treatment groups was significantly lower than that in the infected group during early infection. This suggests that SAM may modulate the expression of PRRs to mitigate excessive immune responses, aligning with findings from research on the antiviral properties of natural compounds (Li et al., 2017; Zhang et al., 2021; Zhou et al., 2021; Zhi et al., 2024). Additionally, previous studies have demonstrated that immune-related gene expression is elevated in tissues with higher viral titers (Chen et al., 2020; Huang et al, 2021). Therefore, the reduced PRRs expression observed in the SAM-treated groups may be a secondary consequence of decreased viral load and/or diminished PAMPs stimulation. Prior research has indicated that targeting TLRs represents a strategy for protecting chickens against AIV infection (Raj et al., 2023). For instance, chickens administered with ligands for TLR3 and TLR21 reduced H9N2 viral shedding. The precise mechanism by which SAM modulates the expression of PRRs in chickens requires further investigation.

Following sensing AIV nucleic acids, host cells initiate the production of type I interferons (IFNs), which establish an antiviral state by inducing the expression of numerous ISGs via the Janus kinase (JAK) and signal transduction and activators of the transcription (STAT) signaling pathway (Kaiser et al., 2001; Stark and Darnell, 2012; Shrestha et al., 2021; Oladokun et al., 2025). In chicken experiments, it has been shown that the H9N2 virus activates innate immune responses, including the production of interferon, pro-inflammatory cytokines, and interleukin, as well as the induction of antiviral proteins (Kaiser et al., 2001; Shrestha et al., 2021; Oladokun et al., 2025). In this study, H9N2 AIV infection induced the expression of IFN-β, OASL, and MX-1 predominantly in the later stages of infection, consistent with the kinetics of LPAIV infection (Haller et al., 2015; Tag-El-Din-Hassan et al., 2017). Notably, SAM treatment significantly enhanced the mRNA expression levels of OASL and MX-1 in the lungs as early as 2 DPI, prior to the peak of IFN-β induction (G1 and G3 groups). OASL contributes to antiviral defence by activating RNase L to degrade viral RNA (Tag-El-Din-Hassan et al., 2017). Previous studies have reported that chicken OASL was significantly upregulated in CEFs and tracheal epithelial cells infected with H1N1 or H7N9 AIV, indicating its antiviral defense against AIV infection (Jang et al., 2015; Zhao et al., 2023). Moreover, as an antiviral effector, the interferon-induced GTP-binding protein MX was upregulated upon AIV infection, with the level of induction correlating with viral pathogenicity (Haller et al., 2015; Saito et al., 2018; Vu et al., 2022). Although viral loads in the lung and spleen did not exhibit significant differences among the four SAM-treated groups, OASL and MX-1 expression were notably upregulated in the G1 and G3 groups during the early stage of infection. This indicates that SAM may accelerate the establishment of an antiviral state, thereby limiting viral replication in the initial phase. This effect, however, was not observed in the G2 and G4 groups. In the later stages of infection, the overall reduction in viral load across all treatment groups likely resulted in diminished PAMPs-mediated stimulation of immune signaling pathways, thereby contributing to the suppression of OASL and other ISGs. In contrast, IFN-β expression was substantially upregulated during the later stages of viral infection, yet remained lower in the SAM-treated groups than in the infected group, suggesting that SAM may modulate the expression of IFN at later stages of infection. Consequently, these findings reveal a dual role for SAM in enhancing early antiviral defenses while mitigating excessive interferon activation during late-stage infection.

In chickens, the kinetics and intensity of inflammatory cytokine induction are directly correlated with the pathogenicity of the AIV strain (Kalaiyarasu et al., 2016; Rebel et al., 2011; Vervelde et al., 2013; Xing et al., 2008). LPAIV infection typically triggers mild or delayed expression of pro-inflammatory cytokines, which is associated with minimal or delayed changes in anti-inflammatory cytokine expression in infected chicken cells and tissues (Kalaiyarasu et al., 2016; Nang et al., 2011; Vervelde et al., 2013). Interestingly, infection with H9N2 AIV does not induce the early expression of key pro-inflammatory cytokines in the lungs of chickens, such as IL-2, IL-6, or IL-8 (Nang et al., 2011), which aligns with the findings of our study. Additionally, a balanced interplay between pro- and anti-inflammatory cytokines is critical for regulating immune cell recruitment and activation, providing feedback control of cytokine production, and managing the acute phase response (Dinarello, 2009; Rebel et al., 2011; Tavares et al., 2017; Vervelde et al., 2013). In this study, treatment with SAM not only effectively suppressed the excessive expression of cytokines but also contributed to restoring their balanced expression, thereby potentially reducing tissue damage. Furthermore, previous studies indicate that SAM may attenuate tissue damage and inflammatory responses through various pathways, such as the TNF/IL-17 axis in lipopolysaccharide-induced acute lung injury and the PERK/STAT3/NF-κB signaling pathways in CA16 infection (Xiao et al., 2018; Wang et al., 2025). The potential involvement of similar mechanisms of SAM in H9N2 infection warrants further investigation. These findings suggest that SAM exerts a dual immunomodulatory effect by suppressing excessive inflammation while modulating antiviral immunity.

In summary, these results indicate that SAM exerts antiviral effects against H9N2 AIV infection by enhancing early ISG expression and attenuating inflammatory cytokine responses during the later stage of infection. Nonetheless, several limitations are presented in this study. First, the expression of immune-relative gene was conducted exclusively at the mRNA level, with protein-level validation not undertaken due to lack of available antibodies. This limitation hindered the direct assessment of immune signaling pathways. Further studies using the knockout models or signaling inhibitors in *vitro* are necessary to elucidate the mechanism of action and therapeutic potential of SAM. Additionally, the replication cycle of the avian influenza virus primarily consists of four stages: adsorption, entry, replication, and release. Although SAM was found to inhibit viral replication, the specific stage at which SAM exerts its antiviral effect remains unclear. Moreover, isolating and identifying the key active components within the SAM extract will be critical for the development of effective adjuvants that enhance the efficacy of existing vaccines.

## Conclusion

This study demonstrates that SAM exhibits antiviral effects against H9N2 AIV infection in SPF chickens. SAM confers protection by inhibiting viral replication and processing a dual immunomodulatory effect that suppresses excessive inflammation and modulates antiviral immunity. These findings underscore the potential of SAM as a candidate for the prevention and treatment of H9N2 AIV infection in poultry, offering a possible alternative or adjunct to current vaccination strategies.

## Data availability

The data will be accessible upon request, and further inquiries can be directed to the corresponding author.

## Authorship contribution statement

Jianni Huang conceived and designed the study and wrote the initial manuscript. Yanjiao Liang and Jingting Yang performed the experiment and collected the original data. Changmao Jian analyzed the data and interpreted the results. Miaoxiang Zhang assisted in study design and provided critical statistical analysis. All authors contributed to drafting the manuscript, critically revised it for important intellectual content, and approved the final version for submission.

## Disclosures

The authors declare the following financial interests/personal relationships which may be considered as potential competing interests: Jianni Huang reports financial support was provided by National Natural Science Foundation of China. Jianni Huang reports financial support was provided by Guangxi Science and Technology Major Program. Jianni Huang reports financial support was provided by Guangxi Natural Science Foundation. Jianni Huang reports financial support was provided by Guangxi Key Research and Development Plan. If there are other authors, they declare that they have no known competing financial interests or personal relationships that could have appeared to influence the work reported in this paper.
